# Prevalence and Predictor of Exclusive Breastfeeding among Mothers of 0 to 6 months Infants from Pastoralists and Hunters' Community in Tanzania; A Community Based Cross-Sectional Study

**DOI:** 10.24248/eahrj.v5i1.654

**Published:** 2021-06-11

**Authors:** Fabiola Vincent Moshi, Esther E. Akyoo, Saada Ally Seif

**Affiliations:** a Department of Nursing Management and Education, School of Nursing and Public Health of University of Dodoma, Tanzania; b Department of Clinical Nursing, School of Nursing and Public Health of University of Dodoma

## Abstract

**Background:**

Initiating breastfeeding during the first hour after birth and continuing breastfeeding exclusively for 6 months prevents childhood infections such as diarrhoea. Exclusive breastfeeding (EBF) for the first 6 months of life of the baby is recognised globally as the best and the most effective intervention to ensure the survival of babies. The aim of this study was to determine the prevalence of EBF and its predictors among mothers of 0 to 6 months infants from pastoralists and hunters’ community in Manyara region-Tanzania.

**Methods:**

This was a community-based analytical cross-sectional study that involved 342 mothers of 0 to 6 months infants who were randomly selected through 4 stage multistage sampling technique. Data was collected using an interviewer-administered questionnaire. Collected data was analysed using Statistical Package for Social Sciences (SPSS) version 20. Binary Logistic Regression analysis was used to establish factors associated with EBF practices.

**Results:**

The prevalence of EBF among postnatal women from hunters and pastoralists societies was 47.1% at 95% CI=41.7%-52.5%. After adjusted for confounders, the predictors of EBF practice were age of infants (0–1 months, AOR = 2.838 at 95% CI = 1.326–6.075, *p=.007*), age of mothers (26-35 years, AOR=1.851 at 95% CI= 1.059–3.234, *p=.031*), Level of education of infants’ mothers (primary education, AOR= 2.374 at 95% CI= 1.321–4.265, *p=.004*) and knowledge on exclusive breast feeding, AOR=2.51 at 95% CI= 1.435–4.393, *p=.001.*

**Conclusion:**

Majority of mothers from pastoralists’ and hunters’ societies were not practising EBF. Predictors of EBF practice were; the age of infants, maternal age, level of education of the mother and knowledge on exclusive breastfeeding. Poor EBF practice was mainly contributed to low level of knowledge about the EBF. The low level of knowledge could have been contributed by poor access to maternal services. Nature of living (lack of permanent settlement) of the study population could have contributed to low access to maternal services. An innovative interventional study is highly recommended to come up with strategies that will improve knowledge on EBF and practice of EBF

## BACKGROUND

Child mortality remains a public health challenge globally. It is estimated that 70% of deaths that occurred between the ages of 0–25 years in 2019 were costly comprised of children below 5 years, amounting to 5.2 Million deaths. The first month of life was the riskiest period for child survival as 47% of below 5 deaths occurred in this period. 28% of below 5 death occurred among children aged 1-11 Months.^1^

Exclusive Breast Feeding (EBF) for the first 6 months of the life of the baby is recognised globally as the best and most effective intervention to ensure the survival of babies.^2^ Globally, about 35% of babies are breastfed exclusively, whereas in sub-Saharan Africa, babies who are breastfed exclusively range between 22% and 33%.^3^

How infant feeding is done in the first 6 months is diverse and is based on geographical, economical and cultural settings whereby the main concern is the time when mothers initiate breastfeeding, complementary breastfeeding, duration of breastfeeding and the age at which mothers wean their infants.^4^ A study conducted in the United States of America (USA) reveals that practicing EBF for not less than the first 6 months can stop over 900 deaths of infants and children which occur yearly in the USA.^5^

The prevalence of EBF of babies from birth to 6 months has however increased among developing countries from 33% in the year 1995 to 39% in the year 2010 and the West and Central Africa have shown much improvement.^6^ Despite so many studies having been conducted on EBF, the practice is still not extensive in developing countries and is still far beyond the global expectation of 90%, which leaves a gap for much betterment. Furthermore, the associated factors can be quite different between one country and another or even within the same country.^7^ In a study conducted in Baghdad, exclusive Breastfeeding is reported to have the ability to decrease the risk of lower respiratory infections. The study further states that the infants who are provided with formula feeding have a 2.7 times risk for Acute Respiratory Infection (ARI) compared to breastfed infants while those infants breastfed for less than 3 months have a 2.5 times risk for ARI.^8^

Milk from a woman's breasts boosts sensory and cognitive maturity and protects the baby from common childhood illnesses and chronic illnesses as well as shortening the time to recover from illness.^9^ Studies have also indicated that breast milk prevents metabolic diseases especially against obesity and type 2 diabetes mellitus.^10^ Early initiation of complementary foods and liquids decrease the amount of breast milk the infant takes and thus reducing the absorption of the necessary nutrients found in breast milk, hence increasing the chances for the infant to acquire ARI and diarrhoea.^11^ Other studies have shown that EBF is associated with a reduced risk of HIV infection from mother to child.^12^

Milk from a woman's breasts is the only food derived from nature that a baby requires for the first 6 months of life. Breast milk is easily digestible and absorbable, provides the baby with all nutrients and contains digestive enzymes. Infants who are breastfed exclusively for 6 months tend to develop a high IQ, less risk for developing childhood obesity and are also protected against childhood diseases.^13^ EBF is protective against otitis media.^14^ EBF also has benefits for the mother whereby; it increases hormone production which helps in the uterine contractions preventing postpartum haemorrhage. It also acts as natural contraception as it delays the time for fertility, reduces the risk for ovarian and breast cancer while promoting bond for both mother and child.^15^

In Tanzania, the EBF rate is still low, at about 59% compared to the global target of 90%, however initiating breastfeeding during the first hour after delivery is at 51%^16^. In the locality of Manyara Region, the EBF is still very low compared to the national level with prevalence of 33.3% according to Tanzania National Nutritional Survey^17^, whereby initiating breastfeeding during the first hour after delivery is reported to be 76%.^16^ In Zanzibar, although mothers have good knowledge of breastfeeding practices, many did not practice EBF and the reported rate of EBF was 20.8%.^18^ This can be explained with the existing challenges for meeting exclusive breastfeeding such as the belief that breast milk alone is not sufficient in meeting babies’ nutritional needs, short maternity leave and socio-cultural pressure to introduce water and artificial feeds.^19^

Previous studies have listed several factors associated with exclusive breastfeeding such as ethnicity of the mother^20^, maternal education^20,21^, family income^20^, perceived inadequate breast milk^21^, maternal age^22^, mode of delivery^21^ and knowledge about EBF.^23^

Despite the sensitisation through media, drama on EBF - practice, still suboptimal breastfeeding is existing in the-Manyara region where the majority of the habitant population are hunters and pastoralists. Little is known on predictors of EBF practices among indigenous pastoralists and hunters’ communities in the Manyara region.

## METHODS

### Research Design

This study was a community-based cross-sectional analytical study that involved assessing the exposure and outcomes ofEBF and predictors of EBF practice.

### Study Area

This study was conducted in rural areas of the Manyara region in 3 districts namely; Mbulu, Simanjiro, and Hanang. This Region is located in the Great Rift Valley and has large herds of cattle and wildlife reserves. It is home for the most distinctive indigenous tribes. The Hadzabe tribe who live in Yaeda Chini valley practice hunting and collecting of wild honey, roots and wild fruits. Other tribes found in the region are; Iraqw, Gorowa, Maasai, Barbaig/Mang’ati.^24^ In a report from the 2012 National Census, the region has a population of 1,425,131 people. Among these, females are 708,046.^25^ The region has 10 hospitals, 25 health centres and 207 dispensaries. Similar to other regions, the numbers of health facilities do not match the population, also, the region has an additional barrier towards accessing maternal health services. The majority of hunters and pastoralists do not have permanent settlements as they migrate depending on the availability of pasture. The majority of rural dwellers in Tanzania depend very much on health facilities as their sole source of health education.

### Study Population

The study population included all mothers from pastoralists and hunters’ community together with their infants of 0 to 6 months of age in the 3 selected districts within the Manyara region.

### Inclusion Criteria

Postnatal mothers with infants aged 0 to 6 months from hunters and pastoralists societies who consented to participate in the study.

### Exclusion criteria

Postnatal mothers with infants aged 0-6 months from hunters and pastoralists societies who were very sick, mentally sick and those with infants who were very sick.

### Sample Size Estimation

This study used the proportion of 33.3% of women practicing EBF in the Manyara Region as reported in the Tanzania National Nutrition Survey.

The formula used was adopted from Cochran, 1975


$$\text{n}=\frac{{\text{Z}}^{2}\text{pq}}{{\text{e}}^{2}}$$

Where;

n= minimal sample size desired

p= proportion of the target population estimated to be exclusively breastfed 33.3%

q=1-p

e=marginal error 5%

Z = standard normal deviation at, 95% confidence interval 1.96

n=1.96^2^ × 0.333 (1-0.333)

0.05^2^

n=323.5 ≈ 324

The computed figure 324 was adjusted by 10% for the inconsistencies and incompleteness. The sample size arrived at was 356 participants.

### Sampling Technique

Manyara Region was selected purposively because it showed a low prevalence of EBF (33.3%) as reported in the Tanzania National Nutrition Survey^17^. It is also the home for pastoralists and hunters community. The study employed the multistage sampling technique. This involved 4 stages:

**First stage:** Selection of Districts: 3 districts out of 5 districts in the Manyara region were randomly selected using Simple Random Sampling by Lottery Selection.

**Second stage:** Selection of Wards: Simple Random Sampling by Lottery method was employed to select 2 wards out of 8 wards in each district, making a total of 6 wards.

**Third stage:** Selection of Villages: On each of the selected ward, 3 villages out of 4 was selected using Simple Random Sampling by Lottery method, making a total of 18 villages. Each ward in Tanzania contains approximately 4 villages.^25^

**Fourth stage:** Selection of Household: After obtaining a list of households with infants from the community health care workers’ register, 19 households out of approximately 34 households were selected using Simple Random Sampling from each of the 18 villages.

### Data Collection Method

Data was collected using interviewer-administered questionnaires and documentary reviews. 5 research assistants were recruited and trained about the tool and data collection process. The principal investigator and the research assistants were fluent in 3 languages; English, Swahili and the local language of the interviewee. The tool was developed in English and translated into Swahili. The questionnaire was then translated to specific local languages to facilitate easy capture of the data. Documentary review involved analysing Reproductive and Child Health (RCH) cards which were obtained from mothers. The instrument was piloted among unselected villagers in Manyara Region and minor modifications were done before the final document of the tool was released. Data was collected from 8th April 2019 to 6^th^ June 2019.

### Variables

Independent variables were maternal characteristics and socio-cultural factors. The dependent variable was prevalence and predictors of EBF

### Variable Measurement

EBF practice was measured by using close-ended questionnaires with 7 selected items each having I mark for the correct answer and 0 score for incorrect answer. Before Principal Component Factor (PCA), analysis was done to extract the items, one item was excluded since it had the same response (yes). On the first phase of PCA, the variance was 33.694 and all remaining 6 items were above 0.3. Mean was 0.86, median 0.97, minimum 2.48 and maximum was 2.85976. Since data was not normally distributed, median was used as a cut-point for categorisation. Participants who scored below-median were considered as non-EBF mothers while those scoring above the median were considered as EBF mothers.

Maternal knowledge of EBF was measured by 22 items. Each right answer had I mark and the wrong answer had a zero mark, making a total of 22 (100%). Principle Factor Analysis was employed to extract items. Among 22 items, 2 items carried the same value (yes) for all respondents so they were excluded during data processing.

In the first phase of Principle Factor Reduction analysis, 20 items were included and the highest variance was 25.737, then 2 items were again excluded because they had a weighted score of <0.3. During the 2^nd^ phase of Factor Reduction, 18 items were selected, the highest variance was 31.011. All the remaining 18 items scored more than 0.3, therefore these items were used for scoring. Normality test showed that data was normally distributed. Therefore, mean was selected to be the cut point for scoring. Mean =0.49, median=0.27, mode=2.72, SD 1.14, minimum −1.75, maximum=2.72. Those who scored above the mean had adequate knowledge, and those who scored below mean had inadequate knowledge.

### Data Analysis

Data was first checked for completeness and missing information and then manually cleaned before it was analysed using Statistical Package for Social Sciences (SPSS) version 20 developed by IBM USA. Demographic characteristics were analysed by Descriptive Statistics to indicate frequencies and percentages. Frequencies were recorded in charts, which showed the prevalence and magnitude of the selected variables. A normality test was used to test the distribution by histogram. To assess the level of knowledge of EBF and practices of breastfeeding among respondents, Principal factor analysis was employed to determine the scores.

A chi-square test was used to test the relationship between maternal variables and EBF practices. Maternal variables with a p-value less than 0.2 in the Chi-square test were entered into a regression model to establish the association. Binary Logistic regression was used to determine the strength of association between the selected variables controlling for confounder variables whereby both Odds Ratio (OR) and Adjusted Odds Ratio (AOR) were reported. Significance level was set to 0.05 *(P <.05*) equivalent to 95% Confidence Interval.

### Ethics Approval and Consent to Participate

The proposal was approved by the Ethical Review Committee of the University of Dodoma in Dodoma, Tanzania Ref: UDOM/DRP/134/VOL VII/36. Furthermore, a letter of permission was obtained from the Manyara Region Administration. Both written and verbal consents were sought from study participants after explaining to them the study objectives, procedures and their right to refuse to participate in the study at any time they wish. For study participants who were younger than 18 years, verbal consent to participate was sought from their parents or guardians.

## RESULTS

### Socio-Demographic Characteristics of the Infants Aged 0-6 Months and their Mothers

Of the 356 sampled mothers, the response rate was 96% which is equivalent to 342 mothers.

The majority, 155(45.3%) of infant mothers were in the age group of 16–25 years with mean 27.59±6.498. Nearly half, 164(48%) of the study respondents had no formal education. The majority of infants were aged between 4–5 months 130(38.0%). The mean age of the infants was 2.76±1.023. Most of the infants, 227(66.4%) were male and most of them 222(64.9%) had good progress in immunisation according to schedule (Table 1).

**TABLE 1:** Socio-Demographic Characteristics of the Infant and Mother (N=342)

Variable	Frequency (n)	Percentage (%)
**The age group of infant mothers**		
16–25 years	155	45.3
26–35 years	137	40.1
36–45 years	50	14.6
**Education of infant mothers**		
None	164	48.0
Primary	154	45.0
Secondary/higher	24	7.0
**Marital status of infant mothers**		
Married/cohabiting	317	92.7
Unmarried	25	7.3
**Occupation of the infant-mother**		
Livestock keeper	151	44.2
Hunter	54	15.8
Peasants	103	30.1
Employed/Self-employed	34	9.9
**Tribe of the infant-mother**		
Maasai	102	29.8
Barbaiq/Mang’ati	86	25.1
Iraqw	79	23.1
Hadzabe	52	15.2
Other tribes	19	5.6
Ndorobo/Akea	4	1.2
**The religion of infant-mother**		
Traditional	168	49.1
Christians	163	47.7
Muslims	11	3.2
**District of residence**		
Mbulu	119	34.8
Hanang	113	33.0
Simanjiro	110	32.2
**Toilet available**		
Yes	326	95.3
No	16	4.7
**Safe water available**		
Safe	98	28.7
Unsafe	244	71.3
**Hygiene**		
Good	304	88.9
Poor	38	11.1
**Parental smoking**		
Yes	127	37.1
No	215	62.9
**The age group of infants (month)**		
0–1	55	16.1
2–3	65	19.0
4–5	130	38.0
6	92	26.9
**Sex**		
Boys	227	66.4
Girls	115	33.6

### Prevalence of Exclusive Breastfeeding Practice

The results of this study show that more than half of the respondents 181 (52.9%) were not practicing EBF while only 161 (47.1%) were practicing EBF. The prevalence of EBF practice was 47.1% and 95% CI= 41.7%-52.5%

### Maternal Knowledge of EBF

In general, among all the study respondents, only 147(43.0%) had adequate knowledge of exclusive breastfeeding

### The Relationship between Socio-Demographic Characteristics and EBF

Socio-demographic characteristics which showed a significant relationship with EBF were the age of the infant *(p<.001),* age group of infants’ mothers (*p=.026*), Mother's level of education (*p<.001*), mother's occupation (*p=.022*), Religion (*p=.005*), mothers’ knowledge about EBF (*p<.001*) and the number of antenatal visits (*p<.001*) (Table 2)

**TABLE 2:** The Relationship between Socio-Demographic and EBF

Variable	EBF(n=145) n(%)	Mixed feeding (n=197) n(%)	χ^2^	*P-value*
**Age group of infants**				
0 to 1 month	22 (40)	33 (60)		
2 to 3 months	36 (55.4)	29 (44.6)		
4 to 5 months	45 (34.6)	85 (65.4)	20.423	<.001
6 months	58 (63)	34 (37)		
**Sex of a child**				
male	104 (45.8)	123 (54.2)		
female	57 (49.6)	58 (50.4)	.431	.512
**The age group of mothers**				
16–25 years	71 (45.8)	84 (54.2)		
26–35 years	74 (54)	63 (46)	7.309	.026
36–45 years	16 (32)	34 (68)		
**Level of Education of mother**				
no formal education	54 (32.9)	110 (67.1)		
primary education	91 (59.1)	63 (40.9)	25.798	<.001
secondary/higher education	16 (66.7)	8 (33.3)		
**Occupation of a mother**				
peasant	49 (47.6)	54 (52.4)		
employed/self-employed	22 (64.7)	12 (35.3)		
livestock keeping	73 (48.3)	78 (51.7)	9.620	.022
hunting	17 (31.5)	37 (68.5)		
**Marital status**				
Married/cohabiting	153 (48.3)	164 (51.7)		
Not married	8 (32)	17 (68)	2.461	.117
**Tribe**				
Iraqw	39 (49.4)	40 (50.6)		
Barbaiq	43 (50)	43 (50)		
Hadzabe	17 (32.7)	35 (67.3)		
Maasai	47 (46.1)	55 (53.9)	11.002	.051
Ndorobo	1 (25)	3 (75)		
Others	14 (73.7)	5 (26.3)		
**Religion**				
Christian	88 (54)	75(46)		
Muslim	8 (72.7)	3(27.3)	10.772	.005
Traditional/pagan	65 (38.7)	103(61.3)		
**Parity**				
Primipara	62(43.7)	80 (56.3)		
Multipara	99 (49.5)	101 (50.5)	1.136	.287
**Number of ANC Visits**				
None	29 (27.1)	78 (72.9)		
One-three	79 (52)	73 (48)	27.976	<.001
Four or more	53 (63.9)	30 (36.1)		
**Place of Childbirth**				
Health Facility	82(41.2)	117(58.8)		
Traditional Birth attendants	99(69.2)	44(30.8)	26.230	<.001
**Knowledge about EBF**				
Adequate	103(65.6)	54 (34.4)		
Inadequate	58(31.4)	127 (68.6)	39.995	<.001

When adjusting for confounders, knowledge on EBF was found to be a strong predictor of EBF. Those with adequate knowledge on EBF were nearly 3 times more likely to have good breastfeeding practices compared to those with inadequate knowledge, AOR=2.51 at 95% CI= 1.435–4.393,*p=.001,* age of infants (6 months, AOR= 2.838 at 95% CI = 1.326–6.075, *p=.007*), age of mothers (26-35 years, AOR=1.851 at 95% CI= 1.059–3.234, *p=.031*) and level of education of infants’ mothers (primary education, AOR= 2.374 at 95% CI= 1.321–4.265, *p=.004*) (Table 3).

**TABLE 3:** Predictors of Exclusive Breastfeeding (EBF) (N=342)

Variable	OR	95% CI		*P-value*	AOR	95% CI		*p-value*
		Lower	Upper			Lower	Upper	
**The age group of infants**								
6 months	1				1			
4 to 5 months	1.86	0.899	3.857	.094	2.097	0.937	4.696	.072
2 to 3 months	0.79	0.415	1.52	.487	1.041	0.506	2.142	.914
0 to 1 months	2.56	1.289	5.08	.007	2.838	1.326	6.075	.007
**The age group of mothers**								
16–25 years	1				1			
26–35 years	1.39	0.876	2.204	1.39	1.851	1.059	3.234	.031
36–45 years	0.56	0.284	1.091	.557	1.985	0.84	4.689	.118
**Level of Education of mother**								
no formal education	1				1			
primary education	2.94	1.862	4.649	.000	2.374	1.32	4.265	.004
secondary/higher educ.	4.07	1.642	10.111	.002	1.864	0.535	6.491	.328
**Occupation of a mother**								
peasant	1				1			
employed/self-employed	2.02	0.905	4.508	.086	0.987	0.351	2.771	.98
livestock keeping	1.03	0.625	1.703	.904	1.14	0.636	2.041	.66
hunting	0.51	0.253	1.012	.054	1.019	0.439	2.366	.964
**Knowledge about EBF**								
Inadequate	1				1			
Adequate	4.18	2.656	6.568	.000	2.51	1.435	4.393	.001
**Number of ANC Visits**								
None	1				1			
One-three	2.91	1.71	4.955	.000	1.554	0.766	3.152	.222
Four or more	4.75	2.561	8.818	.000	1.954	0.843	4.53	.119
**Place of Childbirth**								
Traditional Birth attendants	1				1			
Health Facility	3.21	2.04	5.053	.000	1.445	0.767	2.72	.255

## DISCUSSION

In the current study, the prevalence of EBF among mothers of infants aged 0-6 months from pastoralists and hunters’ communities in the Manyara Region was low (42.4%) if compared to the national average of EBF of 59%.^22^ A higher prevalence of EBF has been reported by a similar study done in Kigoma Tanzania.^27^ The reason for the difference could be due to differences in study settings. The current study was done among rural communities while the study in Kigoma was among urban dwellers. The low prevalence could be due to cultural factors and the nature of lifestyle of this community. A previous study done in Kenya has reported that socio-cultural beliefs (considering colostrum as ‘dirty, a curse, bad omen, associated with breastfeeding while engaging in extramarital affairs, a fear of the ‘evil eye’ when breastfeeding in public) play suboptimal roles in EBF practice.^28^

The hunters and pastoralists do lack permanent settlements, they move according to the availability of pasture and food. For such a lifestyle, it becomes hard for them to access maternal health services where they could access health education on EBF.

The findings from the current study showed that the EBF prevalence (42.4%) was higher compared to the previous study by Tanzania National Nutrition Survey which reported the EBF in Manyara region to be 33.3%^17^. This increase could be because of the time that has passed since the survey was conducted and women may have acquired some knowledge since several media sensitise people about of importance EBF. These results were compared to findings from previous studies in Ghana, where it was reported that the EBF practice was 66.0%, Manhean et al.^29^, this is higher than the current study. The difference could be due to differences in sample characteristics and knowledge the mothers had on EBF and methods used for data collection. Meanwhile, a study in Bangladesh reported that EBF practices were at 35.90%.^30^ However, the findings from another study in Ethiopia^31^ reported EBF practice of 44.2% which was in contrast to the current study. Furthermore, a study which was conducted in rural Coast region Tanzania reported EBF practice to be at about 30%.^32^ However, a study conducted in Muheza reported EBF practice to be 24.1%.^3^ This difference in results could be due to the difference in sample size which was used and geographical location.

The study found that maternal knowledge about EBF significantly influenced their practice on EBF. Postnatal mothers who had adequate knowledge of EBF were almost 3 times more likely to practice Exclusive Breastfeeding when compared with those with inadequate knowledge. This means, knowledge of EBF plays an optimal role in EBF practice. Similar previous studies conducted elsewhere have also reported significant association between knowledge on EBF and EBF practice.^33,34^

In the current study, only 45.9% of postnatal mothers had adequate knowledge of EBF. The reason for inadequate knowledge of EBF could be that women in this community have low level of education. Secondly, because they move from place to place grazing livestock, they have little time to attend ANC from where they could get knowledge of EBF. Another reason could be due to the language used to provide health education during maternal services visits at health centres, the majority of women of this community could only understand messages spoken in their local languages while the medium of communication in health facilities is Swahili.

In contrast, a similar study in Nigeria reported low knowledge of EBF where only 30.0% of women were adequately informed.^34^ However, in a study conducted in Ghana among rural lactating mothers, 74% had general knowledge of EBF which they got from health care providers during antenatal and postnatal visits.^35^

In Malawi, the rate of EBF among babies below 6 months of age increased from 3% in 1992 to 71% in 2010. This was attributed to the government's implementation of the Infant and Young Child Feeding (IYCF) program as well as enrolment of a mass education program to increase support and EBF knowledge.^11^

The study also found that the age of the infant influenced EBF practice. Postnatal mothers with neonates were almost 3 times more likely to practice EBF compared to postnatal mothers with infants aged 6 months and above. The reason for this could be due to the belief that breast milk alone cannot meet the nutritional requirements of older infants. Also, this could be due to maternal engagement in household activities which may sometimes necessitate separation with the infant for hours. Similar previous studies on predictors of EBF have reported similar findings.^19^-^33^

The study also found that maternal level of education predicted significantly EBF practice. Postnatal mothers who had primary education were twice more likely to practice EBF compared to postnatal mothers with no formal education. A previous study conducted in Northern Tanzania on predictors of EBF practice reported a different finding that women's level of education does not predict the practice of EBF.^3^ Different findings could be due to differences in the study communities. In the current study-language barrier could have played a great role in hindering acquisition of knowledge on EBF. Postnatal mothers who attended primary level of education have the advantage of learning the Swahili Language, the language used in the delivery of maternal health services-including health education in health centres. This factor could have favoured them to acquire adequate knowledge on EBF which in turn facilitated the practice of EBF. The current study also found that maternal age predicted the practice of EBF. Postnatal mothers with advanced age were twice more likely to practice EBF than young postnatal mothers. The finding is in line with a similar previous study conducted in Northern Tanzania.^3^ Another study conducted in Zimbabwe has reported maternal age of below 25 years as a barrier towards EBF practice.

### Study Limitations

The key information gathered from the study participants was self-reported which is subject to under-or over-reporting. Despite the systematic data collection approach, there may have been some intrinsic bias in the questionnaire or manner in which questions were asked that might have affected the responses. Although the reported predictors were controlled through regression analysis, the causal-effect relationship study is recommended.

## CONCLUSIONS

The EBF practice is poor in the Manyara region. Postnatal mothers who were more likely to practice EBF were those of advanced age, with infants aged 0–1 months, had a primary level of education and had an adequate level of knowledge on EBF. The study recommends an innovative interventional study to come up with cost-effective strategies to improve EBF practice among hunters and pastoralists communities, specifically, to address their knowledge on EBF as the vast majority of them had inadequate knowledge on EBF.
